# Edmonton frailty scale score predicts postoperative delirium: a retrospective cohort analysis

**DOI:** 10.1186/s12877-022-03252-8

**Published:** 2022-07-15

**Authors:** Frederick Sieber, Susan Gearhart, Dianne Bettick, Nae-Yuh Wang

**Affiliations:** 1 Department of Anesthesiology and Critical Care Medicine, Johns Hopkins Medical Institutions, Johns Hopkins Bayview Medical Center, 4940 Eastern Avenue, Baltimore, MD 21224 USA; 2 Department of Surgery, Johns Hopkins Medical Institutions, Baltimore, MD USA; 3 Johns Hopkins Bayview Medical Center, Baltimore, MD USA; 4 Departments of Medicine (General Internal Medicine), Biostatistics, and Epidemiology, Johns Hopkins Medical Institutions, Baltimore, MD USA

**Keywords:** Frailty, Delirium, Complications, Postoperative, Elective surgical procedures

## Abstract

**Background:**

Frailty has been associated with postoperative delirium (POD). Studies suggest that the Fried phenotype has a stronger association with POD than the Edmonton Frailty Scale (EFS) criteria. Although phenotypic frailty is recognized as a good predictor of delirium, the EFS has higher ratings for feasibility in the surgical setting. Thus, our aim was to determine the association between EFS-assessed vulnerability and POD in an elective surgical population of older adults. A secondary aim was to determine which domains assessed by the EFS were closely associated with POD.

**Methods:**

After IRB approval was received, electronic medical records of surgical patients at our institution were downloaded from 12/1/2018 to 3/1/2020. Inclusion criteria included age ≥ 65 years, preoperative EFS assessment within 6 months of surgery, elective surgery not scheduled for intensive care unit (ICU) stay but followed by at least 1 day postoperative stay, and at least two in-hospital evaluations with the 4 A’s test (arousal, attention, abbreviated mental test-4, acute change [4AT]) on the surgical ward. Vulnerability was determined by EFS score ≥ 6. Patients were stratified into two groups according to highest postoperative 4AT score: 0–3 (no POD) and ≥ 4 (POD). Odds of POD associated with EFS score ≥ 6 were evaluated by using logistic regression adjusted for potential confounders.

**Results:**

The dataset included 324 patients. Vulnerability was associated with higher incidence of POD (*p* = 0.0007, Fisher’s exact). EFS ≥6 was consistently associated with POD in all bivariate models. Vulnerability predicted POD in multivariable modeling (OR = 3.5, 95% CI 1.1 to 11.5). Multivariable analysis of EFS domains revealed an overall trend in which higher scores per domain had a higher odds for POD. The strongest association occurred with presence of incontinence (OR = 3.8, 95% CI 1.2 to 11.0).

**Conclusions:**

EFS criteria for vulnerability predict POD in older, non-ICU patients undergoing elective surgery.

**Supplementary Information:**

The online version contains supplementary material available at 10.1186/s12877-022-03252-8.

## Background

Frailty, as determined by either phenotypic or deficit accumulation instruments, has been associated with a higher incidence of post-operative delirium (POD), defined as an acute confusional state characterized by inattention, abnormal level of consciousness, thought disorganization, and a fluctuating course that happens after an older adult has an operation (surgery) [1]. Indeed, a recent meta-analysis that compared POD incidence in frail versus non-frail older patients undergoing elective surgery supported these findings, reporting an adjusted odds ratio (OR) estimate of 2.14 (95% confidence interval [CI] = 1.43–3.19) [2]. However, this meta-analysis combined studies that used either phenotypic or deficit accumulation criteria. In addition, the range of POD incidence for the included studies was 7 to 56% owing to differences in patients, surgical procedures, and surgical risks [2]. Although frailty is generally recognized as a predictor of delirium, it is less clear what POD risk level is associated with frailty for older patients undergoing in-patient elective surgeries that do not require postoperative intensive care unit (ICU) care.

Many frailty assessment tools, both physical phenotype and deficit accumulation, strongly predict POD in older adults [3]. Recent meta-analysis suggests that the physical frailty phenotype has the strongest association with POD [4]. However, that study was not a head-to-head comparison and was underpowered, but it supports the notion that deficit accumulation frailty screening tools, such as the frailty index (FI) or Edmonton Frailty Scale (EFS), may underestimate the association between frailty and POD. Therefore, another open question is whether frailty screening with deficit accumulation instruments is associated with POD risk in lower-risk surgical settings.

On the other hand, deficit accumulation frailty instruments, such as FI and EFS, have predominately positive feasibility ratings compared to the frailty physical phenotype [4], and are commonly used in geriatrics to screen for underlying vulnerability [5]. Practitioners need an easy screening test for frailty or high-risk conditions that would alert the perioperative team to perform a more rigorous evaluation in the form of a comprehensive geriatric assessment. The FI and EFS are comparable in both feasibility and their association with POD [4]. However, EFS does require training for measurement of its physical components. At our institution EFS is used in the surgical clinics for preoperative frailty screening. This decision was based on the difference in reported time requirement for completion of EFS (< 5 min) vs FI (10–12.5 min) [4].

The EFS assesses for multiple domains including cognition, hospital admissions and general health, ADL needs, social support, polypharmacy and forgetting to take medications, weight loss, depression, incontinence, and level of function [5]. Although the EFS does not specifically assess for delirium risk, the American College of Surgeons best practice guidelines for geriatric patient care recommend screening for several POD risk factors contained within the EFS domains [6]. Because the EFS assesses for many of the delirium risk factors contained in the American College of Surgeon’s screening recommendations, we hypothesize that vulnerability, as detected by the EFS, will be an independent risk factor for POD. Given its feasibility and use in our clinical practice, we wanted to know whether EFS-determined vulnerability is a predictor of POD risk in older patients undergoing lower-risk surgery. Our aim was to determine the association between EFS-assessed vulnerability and POD in a sample of older, non-ICU patients undergoing lower-risk elective surgery. A secondary aim was to determine which domains assessed by the EFS were closely associated with POD in this population.

## Methods

After receiving IRB approval, which included a waiver of consent requirements, we downloaded data from the electronic medical record (EMR) for patients who underwent surgery at our institution between December 1, 2018, and March 1, 2020. During this time period 83 eligible vulnerable subjects (EFS ≥ 6) were identified. Downloads contained data on demographics, medication usage, vital signs, and nursing documentation. The data security protocol was reviewed and approved by the institutional Data Trust.

The following inclusion criteria were used for this retrospective cohort analysis:

- Age ≥ 65 years at the time of surgery
- Preoperative EFS assessment within 6 months of surgery (Note: EFS is a licensed product; waiver for use was granted by D. Rolfson)
- Elective surgery
- Initial post-anesthesia recovery in the post-anesthesia care unit (PACU)
- Not initially scheduled for postoperative intensive care. However, patients who were admitted to the ICU later in their hospital stay were included.
- At least 1 overnight stay on the surgical ward immediately after PACU discharge.
- At least two in-hospital evaluations with the 4 A’s test© [7] (arousal, attention, abbreviated mental test-4, acute change [4AT]) during the patient’s stay on the surgical ward. (Note: The 4AT policy allows free downloads, use, and copying as required for non-commercial or research use).

Since March 1, 2018, patients ≥65 years of age in the surgical clinics at our institution have undergone preoperative screening for vulnerability with the EFS. To implement EFS screening, all surgery clinic nursing personnel (*n* = 18) underwent standardized in-service training for EFS administration followed by competency testing. A patient with an EFS score ≥ 6 is defined as vulnerable. Since December 1, 2018, the nursing staff has documented in-hospital delirium assessments on the surgical ward at least once every 12 hours using the 4AT score in all surgical patients ≥65 years of age. At our institution, a 4AT score ≥ 4 after surgery is considered a positive screen for POD. The 4AT has been validated as a screening instrument for delirium with 84.9% specificity and 89.7% sensitivity on the hospital ward [7] and 99.2% specificity and 95.5% sensitivity in the PACU [8]. In our study population, a small number of patients with prolonged hospital stays were admitted to the ICU after their initial PACU and ward admission. In these cases, the CAM-ICU© score [9], routinely obtained by the ICU nursing staff each 8-hour shift, was included in the analysis. EFS, 4AT, and CAM-ICU scores, as well as their individual components, are documented in the EMR. (Note: CAM-ICU Copyright© E.Wesley Ely,MD, MPH and Vanderbilt University, all rights reserved, use is unrestricted and does not require written permission).

### Statistical analysis

Baseline characteristics of eligible patients were described as frequency count and percentage for categorical variables, and as mean and standard deviation (SD), and as median and interquartile range (IQR) when informative, for continuous variables. Patients were stratified into two groups according to highest postoperative 4AT score: 0–3 and ≥ 4, during PACU stay and postoperative hospitalization. Associations between baseline characteristics and 4AT categories were evaluated with a chi-square test or Fisher’s exact test as appropriate for categorical variables, and analysis of variance F-test or Kruskal Wallis test as appropriate for continuous variables. We used penalized likelihood [10] based logistic regression analyses to evaluate the association of EFS score ≥ 6 with the outcome of 4AT ≥4 during hospital stay while adjusting for potential confounders. POD confounders used in logistic regression were taken from the literature and included sex, race, age, American Society of Anesthesiologists (ASA) physical status score, and Elixhauser comorbidity scores for 30-day readmission and in-hospital mortality [11]. Levels of surgical stress were inferred from perioperative blood transfusion totals and total anesthesia time and were included as potential confounders in the regression models. Total anesthesia time was calculated as the summation of anesthesia times for all operating room procedures during the index hospitalization. Elixhauser comorbidity 30-day readmission and in-hospital mortality scores were calculated according to the Agency for Healthcare Research and Quality (AHRQ) Healthcare Cost and Utilization Project (HCUP) instructions (hcup-us.ahrq.gov). We also evaluated the association of 4AT ≥4 during hospital stay with length of hospital stay, hospital discharge disposition, and 30-day mortality after hospital discharge. We carried out additional multivariable logistic modeling to determine relationships between EFS domains and POD.

### Power analysis

Based on our practice volume and past data, it seemed feasible to enroll 320 eligible patients. Targeting this sample size, and assuming that 25% (*n* = 80) would have an EFS score ≥ 6, we calculated that this study would have 85% power to detect a between-group difference of 25% in-hospital incidence of 4AT ≥4 in the EFS ≥6 group versus 10% in-hospital incidence of 4AT ≥4 in the EFS < 6 group (i.e., OR = 3) using a 2-sided z-test with type I error of 0.05.

## Results

**Fig. 1:**
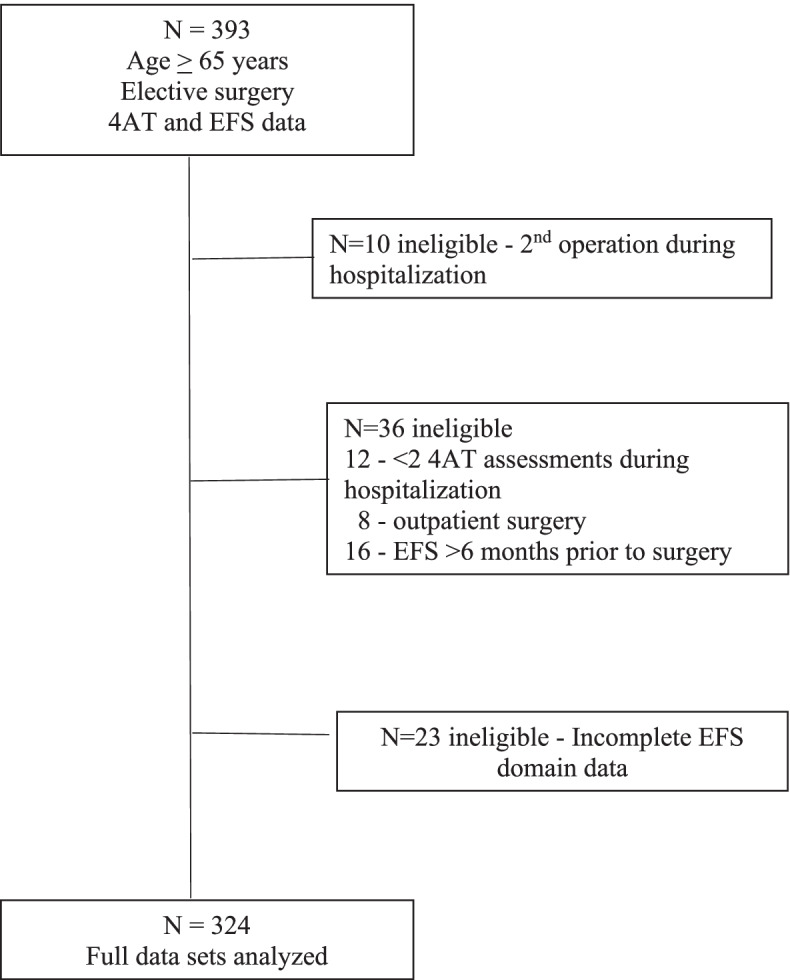
Flow diagram for selection of final electronic medical records for data analysis

During the time period analyzed, 393 patients ≥65 years underwent elective procedures that required at least 1 overnight non-ICU stay and were screened preoperatively for EFS. Of these 393 subjects, 324 had complete EMR datasets (Fig. 1). Our sample included 83 (25.6%) vulnerable patients (EFS ≥6), of which 10 (12.1%) had post operative 4AT scores ≥4. Of the 241 (74.4%) patients without vulnerability, 5 (2.1%) had post operative 4AT scores ≥4 (*p* = 0.0007, Fisher’s exact).

In univariate analysis (Table 1), a higher incidence of 4AT scores ≥4 was associated with increased age and greater comorbidity, as reflected in both the higher ASA score and greater Elixhauser 30-day readmission score.

**Table 1:** Univariate analysis of 4AT score risk factors and correlates among 324 older patients

Parameter	4AT < 4	4AT ≥ 4	*P*
Total, n (%)	309 (95.4)	15 (4.6)	
Demographics			
Sex, n (%)			0.79
Female	123 (96.1)	5 (3.9)	
Male	186 (94.9)	10 (5.1)	
Race, n (%)			0.43
Caucasian	238 (95.8)	10 (4.2)	
Black	61 (92.4)	5 (7.6)	
Other	20 (100.0)	0 (0.0)	
Age, y			
Mean (SD)	73.1 (6.2)	77.5 (6.3)	0.007
Median (IQR)	72.0 (68.0–77.0)	79.0 (71.0–82.0)	0.17
Surgical characteristics and comorbidities			
ASA status, n (%)			0.02
2	92 (97.9)	2 (2.1)	
3	197 (95.2)	9 (4.4)	
4	20 (87.0)	4 (16.7)	
Total anesthesia time, min			
Mean (SD)	237.7 (117.7)	241.8 (147.8)	0.90
Median (IQR)	207.0 (160.0–207.0)	209 (83.0–373.0)	0.79
Total units RBC infused			
Mean (SD)	0.1 (0.5)	0.3 (0.6)	0.12
Median (IQR)	0.0 (0.0–0.0)	0.0 (0.0–0.0)	0.0003
Elixhauser 30-day readmission score			
Mean (SD)	21.7 (23.5)	58.3 (43.4)	0.0056
Median (IQR)	15.0 (5.0–29.0)	55.0 (17.0–92.0)	0.003
Elixhauser mortality score			
Mean (SD)	4.4 (10.2)	13.9 (18.9)	0.001
Median (IQR)	3.0 (−1.0–8.0)	10.0 (1.0–30.0)	0.06
EFS total score ≥ 6, n (%)			0.0007
No	236 (97.9)	5 (2.1)	
Yes	73 (88.0)	10 (12.1)	
Anesthesia type, n (%)			0.07
Regional	23 (95.8)	1 (4.2)	
MAC	13 (81.3)	3 (18.8)	
Spinal/epidural	26 (100.0)	0 (0.0)	
General	247 (95.7)	11 (4.3)	
Surgical service area, n (%)			0.09
General	141 (97.9)	3 (2.1)	
Neurosurgery	12 (92.3)	1 (7.7)	
Orthopedics	30 (100.0)	0 (0.0)	
Urology	35 (92.1)	3 (7.9)	
Vascular	80 (92.0)	7 (8.1)	
Other^a^	11 (91.7)	1 (8.3)	
Characteristics at or after hospital discharge			
Length of stay, days			
Mean (SD)	3.9 (4.4)	8.0 (10.8)	0.0015
Median (IQR)	2.3 (1.3–4.4)	3.9 (1.4–9.2)	0.79
Discharge disposition, n (%)			< 0.0001
Expired	0 (0.0)	3 (100.0)	
Home- / self- / home-health-care	264 (98.1)	5 (1.9)	
Skilled nursing facility	40 (87.0)	6 (13.0)	
Rehab/other	5 (83.3)	1 (16.7)	
30-Day mortality, n (%)			0.0003
No	308 (96.3)	12 (3.8)	
Yes	1 (25.0)	3 (75.0)	

*ASA* American Society of Anesthesiologists, *EFS* Edmonton Frailty Scale, *IQR* Interquartile range, *MAC* Monitored anesthesia care, *RBC* Red blood cells, *SD* standard deviation

^a^Other includes gynecologic, otolaryngologic, and plastic and reconstructive surgeries

In a series of bivariable analyses for 4AT score ≥ 4, predictors incorporated EFS ≥6, and an additional significant univariate predictor showed that EFS was consistently associated with 4AT score ≥ 4 in all bivariable models (Table 2). Age was not significant when adjusted for EFS. However, both measures of comorbidity (ASA and Elixhauser) maintained significance after EFS adjustment. Only EFS and Elixhauser maintained significance in multivariable modeling.

**Table 2:** Adjusted odds ratios of postoperative 4AT score ≥ 4 associated with selected preoperative risk factors

Models and variables	Odds ratio	95% Confidence limits		*P*
**Bivariable models**				
Model 1				
EFS score ≥ 6, yes vs no	4.86	1.62	14.58	0.005
Age, per 5-year increase	1.37	1.14	1.54	0.09
Model 2				
EFS score ≥ 6, yes vs no	5.25	1.74	15.88	0.003
ASA				0.08^a^
2 vs 4	0.24	0.04	1.35	0.10
3 vs 4	0.24	0.07	0.87	0.03
Model 3				
EFS score ≥ 6, yes vs no	3.86	1.26	11.80	0.018
Elixhauser 30-day readmission score, per 10-point increase	1.32	1.14	1.53	0.0002
**Multivariable model**				
EFS score ≥ 6, yes vs no	3.49	1.06	11.54	0.04
Age, per 5-year increase	1.41	0.95	2.09	0.09
ASA				0.33^a^
2 vs 4	0.67	0.09	5.22	0.70
3 vs 4	0.35	0.08	1.59	0.17
Elixhauser 30-day readmission score, per 10-point increase	1.27	1.07	1.50	0.007

*ASA* American Society of Anesthesiologists, *EFS* Edmonton Frailty Scale

^a^Type 3 Wald’s chi-square test

As shown in Table 1, length of stay (LOS) was greater among patients with 4AT score ≥ 4. Among the 250 patients who had LOS < 5 days, 62 were frail, and 5 of those (8.1%) screened positive for POD during their hospital stay. In contrast, 4 of 188 (2.1%) who were not frail before the surgery screened positive for POD (OR = 3.92; 95% CI: 1.08 to 14.21; *P* = 0.037). LOS did not modify the association between POD and frailty. Patients with 4AT score ≥ 4 were more likely to have requirements for skilled nursing or rehabilitation facilities on hospital discharge.

Four of the 324 patients died within 30 days postoperatively. Thirty-day mortality was associated with 4AT score ≥ 4 as well as increased Elixhauser mortality score.

### Analysis of specific EFS domains

We conducted multivariable analysis of each EFS domain and adjusted for age, ASA, and Elixhauser comorbidity score (Table 3). The data showed an overall trend in which higher scores per domain had a higher odds ratio for postoperative 4AT scores ≥4. Strong associations occurred with presence of incontinence, timed get up and go, decreased functional independence, previous hospital admissions, and forgetting to take medications. Of interest, difficulty with clock draw did not have as strong an odds ratio estimate, although the 95% confidence intervals for these association estimates were generally wide.

**Table 3:** Odds ratios of specific Edmonton Frailty Scale domains for postoperative 4AT score ≥ 4

Edmonton Frailty Scale domain	Odds ratio [95% CI]^a^	*P*
Clock draw		0.44^b^
Spacing errors vs no error	1.27 [0.32–5.00]	0.73
Other errors vs no error	2.62 [0.60–11.42]	0.20
Hospital admissions		0.15^b^
1–2 vs 0	3.02 [0.80–11.33]	0.10
≥ 2 vs 0	3.83 [0.87–16.83]	0.08
General health		0.44^b^
Fair vs good to excellent	0.68 [0.19–2.48]	0.56
Poor vs good to excellent	1.86 [0.41–8.43]	0.42
Functional independence (ADL needs)		0.07^b^
2–4 vs 0–1	3.37 [1.04–10.98]	0.04
5–8 vs 0–1	5.02 [0.84–30.13]	0.08
Social support		0.84^b^
Sometimes vs always	0.72 [0.04–12.80]	0.82
Never vs always	2.63 [0.08–91.68]	0.59
Polypharmacy, yes vs no	0.80 [0.21–3.11]	0.75
Forgetting to take medications, yes vs no	2.89 [0.94–8.82]	0.06
Weight loss, yes vs no	1.43 [0.45–4.55]	0.54
Depressed mood, yes vs no	1.27 [0.37–4.31]	0.70
Incontinence, yes vs no	3.77 [1.19–11.92]	0.02
Functional performance (timed get up and go)		0.10^b^
11–20 sec vs 0–10 sec	3.58 [0.98–13.15]	0.05
> 20 sec / unwilling / needs assistance vs 0–10 sec	3.84 [0.95–15.55]	0.06

*ADL* Activities of daily living

^a^Estimates from separate logistic regression analysis models adjusted for age, ASA status, and Elixhauser comorbidity score for 30-day readmission

^b^Type 3 Wald’s chi-square test

## Discussion

This study shows that EFS-determined vulnerability is a predictor of postoperative in-hospital 4AT scores ≥4 in older non-ICU patients undergoing elective surgery. Among the EFS domains, the strongest associations with in-hospital 4AT scores ≥4 were requirements for assistance with activities of daily living, presence of incontinence, difficulty with timed get up and go, and forgetting to take medications. EFS criteria for vulnerability are predictors of POD in older surgical patients undergoing lower-risk procedures.

In general, most frailty instruments demonstrate associations between frailty and poorer outcomes [12]. For instance, frailty instruments add predictive value for death, new disability, and LOS after major elective surgery [13]. However, surgical outcome studies vary considerably in both type of frailty instrument used and the frailty incidence detected. Unfortunately, there are few head-to-head comparisons of frailty instruments in terms of their ability to predict POD. Data from a meta-analysis that compared EFS and Fried criteria suggested stronger associations for POD with the Fried criteria [4]. In studies providing area under the curve (AUC) data, models report an AUC of 0.695 [14] using the modified Fried criteria, whereas a study using the Groninger criteria reported an AUC of 0.89 [15]. Our bivariate analysis adjusted for Elixhauser comorbidity score and EFS had an AUC of 0.796. Our multivariate analysis adjusted for age, ASA category, Elixhauser comorbidity score, and EFS had an AUC of 0.833. Both of our models that incorporated EFS demonstrated excellent predictive capability for delirium using the EFS ≥ 6 cutoff.

Our study population varied considerably from those in earlier reports. Patients requiring ICU admission or urgent/emergent surgery were excluded. In addition, we included a broad range of surgical specialties. Emergency/urgent surgery, ICU admission, and procedures with high cardiac risk are all strong risk factors for delirium [16]. Their elimination from the study population accounts for the lower delirium case index. Our power calculations assumed a higher incidence rate than we observed. However, the observed odds ratio was higher, which is consistent with our findings that EFS-determined vulnerability is associated with 4AT ≥4. The lower case index and its associated issue of power is likely important. However, the direction of the frailty effect was as expected. Longer length of hospital stay was associated with 4AT ≥4 and higher Elixhauser comorbidity score, but it did not affect the association between POD and frailty. In any case, the strong association that we found emphasizes that use of EFS criteria still gives strong predictive value for POD in older surgical patients undergoing lower-risk procedures.

We wish to drill down on the frailty-delirium relationship by dissecting which EFS items are most closely associated with delirium. On the one hand, the questions on the EFS are not granular. But overall, most EFS domains showed a trend toward predicting delirium. This trend probably accounts for the strong relationship between EFS-detected vulnerability and delirium. The EFS domains that focused on function and mobility had strong associations with delirium and are consistent with those in the literature [16]. Urinary incontinence and delirium might be linked via need for anticholinergic administration or underlying cognitive dysfunction. However, chi-square analysis of anticholinergics with and without incontinence did not show statistical significance. Incontinence is also associated with presence of underlying neurologic disease. However, chi-square analysis of neurologic comorbidities from the Elixhauser score (paralysis, dementia, psychosis) with and without incontinence showed no significance. Thus, the association of the incontinence EFS domain with delirium occurs via some other mechanism.

Additional clinical importance of our study comes from our 30-day mortality predictor analysis (Supplemental table). In multivariate analysis, only postoperative 4AT scores ≥4 were predictive of 30-day mortality. The fact that only four deaths occurred within 30 days of surgery limits the generalizability of this result and accounts for the wide confidence intervals. Nonetheless, future studies on preventing delirium with interventions focused on delirium risk factors, such as frailty, may be important for decreasing 30-day mortality even in older populations undergoing low-risk surgery.

### Strengths and limitations

Here we studied pragmatic frailty and delirium assessments. Both the EFS and 4AT are easily administered and feasible in preoperative surgical clinics and on surgical wards. Full EMR datasets were analyzed, providing opportunities for implementation of EMR-driven quality improvement dashboards. All types of elective non-ICU–requiring surgeries were included, giving a broader base to our understanding of frailty–delirium relationships. Nevertheless, some limitations must be considered. The study was retrospective in an in-hospital setting. Total anesthesia time is an imperfect indicator of surgical stress as some surgeries may take disproportionately longer, but are not necessarily more invasive. POD was assessed in a clinical setting, not up to the gold standard used in a research setting, with an implicit assumption of patients not having surgery-related POD after hospital discharge. Mild cognitive dysfunction may have been underrecognized as the clock draw is limited in its recognition of very mild dementia [17].

Not all wards accepting postoperative patients performed the 4AT and not all surgical clinics performed the EFS assessment preoperatively which limited our sample size and generalizability. The targeted sample size in power evaluation was based on feasibility determined by the past patient volume, but POD event rate in the evaluation was assumed according to general estimates and was too high in light of the lower surgical risk in the population being studied. The study had limited sample size resulting in a small number of cases (number of patients with 4AT score ≥ 4). We were careful in our analyses not to overfit the regression model for a small sized sample, for example, by conducting the adjusted analysis in a sequential way where we first adjusted for one relevant covariate at a time only (model 1 to 3 in Table 2). To address small sample bias in logistic regression analysis, we did use penalized likelihood approach [10] for our analyses. The limited sample size and lower number of events did not allow for extensive multivariable modeling; therefore, residual confounding cannot be ruled out. The small sample size also resulted in less precise association estimate, as apparent in the wide 95% confidence intervals for the odds ratio estimate. However, the results are in keeping with previous meta-analysis results. It is also worth noting that the OR estimate of 3.5 associated with EFS ≥ 6 from the multivariable model reported in Table 2 may represent a weighted average of association levels in the range of higher EFS scores observed in our study. In an exploratory analysis using a two-linear-segment logistic regression model, the OR estimate was 1.0 (0.6 to 1.5) per 1 point increase in EFS score between score of 0 and 5, and 1.8 (1.2 to 2.9) per 1 point increase in EFS score ranged from 6 to 9, the upper limit of EFS score observed in our sample (data not shown). Larger datasets from additional research will be needed to analyze the association using EFS as an ordinal variable that it is, and further examine the association with POD beyond the EFS range observed in our data.

## Conclusion

This study shows that vulnerability, as determined by an EFS score ≥ 6, is a strong predictor of POD in older elective surgical patients who do not require ICU admission. When used as a screening instrument for frailty, the EFS can help the provider detect subtle differences and refer these patients for further diagnostic workup. Thus, the EFS could be considered as an important preoperative assessment tool for determining POD risk in lower-risk surgical populations.

## Supplementary Information

**Additional file 1.**

## Data Availability

The datasets used and/or analyzed during the current study are available from the corresponding author on reasonable request.

## Abbreviations

4AT: 4 A’s test
ASA: American Society of Anesthesiologists
AUC: Area under the curve
CI: Confidence interval
EFS: Edmonton Frailty Scale
EMR: Electronic medical record
ICU: Intensive care unit
IQR: Interquartile range
OR: Odds ratio
PACU: Post-anesthesia care unit
POD: Postoperative delirium
SD: Standard deviation
