# Supplementary material for: Edmonton frailty scale score predicts postoperative delirium: a retrospective cohort analysis
Source: BMC Geriatr. 2022 Jul 15;22:585. doi: 10.1186/s12877-022-03252-8 (PMC9288043; doi:10.1186/s12877-022-03252-8)
Supplement: Supplementary file 1 — Additional file 1. [file 12877_2022_3252_MOESM1_ESM.docx]

**Supplemental Table.** Logistic regression results for predictors of 30-day mortality

| **Variable** | **Unadjusted** | | | | **Adjusted for Elixhauser Mortality Score** | | | |
| --- | --- | --- | --- | --- | --- | --- | --- | --- |
|  | **Odds ratio** | **95% Confidence limits** | | ***P*** | **Odds ratio** | **95% Confidence limits** | | ***P*** |
| Edmonton Frailty Scale score ≥6 (yes vs no) | 2.94 | 0.50 | 17.40 | 0.23 |  |  |  |  |
| Sex (female vs male) | 1.54 | 0.26 | 9.05 | 0.63 |  |  |  |  |
| Race |  |  |  | 0.88 |  |  |  |  |
| Black vs White | 1.54 | 0.22 | 10.76 | 0.66 |  |  |  |  |
| Other vs White | 1.64 | 0.08 | 35.11 | 0.75 |  |  |  |  |
| Age, per 5-year increase | 0.98 | 0.48 | 2.00 | 0.95 |  |  |  |  |
| ASA |  |  |  | 0.31 |  |  |  |  |
| 2 vs 4 | 0.25 | 0.02 | 2.61 | 0.25 |  |  |  |  |
| 3 vs 4 | 0.19 | 0.02 | 1.56 | 0.12 |  |  |  |  |
| Total anesthesia time, per 30-min increase | 1.18 | 1.02 | 1.36 | 0.02 | 1.12 | 0.96 | 1.31 | 0.15^a^ |
| Total units of RBC, per 2-unit increase | 3.20 | 1.04 | 9.83 | 0.04 | 2.08 | 0.66 | 6.55 | 0.21^b^ |
| Elixhauser mortality score, per 10-point increase | 2.73 | 1.54 | 4.86 | 0.0006 |  |  |  |  |
| Postoperative 4AT score: ≥4 vs 0-3 | 57.59 | 7.64 | 434.14 | 0.0003 | 27.11 | 3.18 | 231.32 | 0.003^c^ |

ASA, American Society of Anesthesiologists; RBC, red blood cells.

^a^*P*-value for Elixhauser In-Hospital Mortality Score = 0.002.

^b^*P*-value for Elixhauser In-Hospital Mortality Score = 0.001.

^c^*P*-value for Elixhauser In-Hospital Mortality Score = 0.056.
